# *Kcnn4* Knockout Induces Salivary Gland Fibrosis, Immune Cell Infiltration, and B‐Cell Dysfunction in Mice

**DOI:** 10.1111/cbdd.70412

**Published:** 2026-10-11

**Authors:** Yuan Zhang, Siming Zheng, Degui Jiang, Cuiyun Cui, Xuan Zhang, Hanlin Tao, Gongbo Fu, Linna Cheng, Shichao Duan

**Affiliations:** ^1^ Department of Clinical Laboratory Henan Provincial People's Hospital, People's Hospital of Zhengzhou University, and People's Hospital of Henan University Zhengzhou China; ^2^ Department of Hematology The Affiliated Cancer Hospital of Zhengzhou University & Henan Cancer Hospital Zhengzhou China; ^3^ Academy of Medical Science, Tianjian Laboratory of Advanced Biomedical Sciences Zhengzhou University Zhengzhou China; ^4^ Department of Transfusion Medicine Henan Provincial People's Hospital, People's Hospital of Zhengzhou University, and People's Hospital of Henan University Zhengzhou China; ^5^ Henan Provincial People's Hospital, Henan Eye Hospital, Henan Eye Institute Zhengzhou University People's Hospital, Henan University People's Hospital Zhengzhou China; ^6^ Department of Oncology, Eastern Hepatobiliary Surgery Hospital Naval Medical University Shanghai China; ^7^ Institute of Hematology People's Hospital of Zhengzhou University, Henan Provincial People's Hospital, School of Clinical Medicine, Henan University Zhengzhou Henan China

**Keywords:** fibrosis, IgG deficiency, inflammatory proteomics, Kcnn4 (KCa3.1), lymphocyte infiltration, salivary gland

## Abstract

Kcnn4 (KCa3.1) is a calcium‐activated potassium channel involved in cell volume regulation and immune cell function, but its role in salivary gland homeostasis remains poorly understood. In this study, we generated constitutive Kcnn4 knockout (*Kcnn*4^−/−^) mice to investigate the impact of Kcnn4 loss on salivary glands and systemic inflammation. Histological examination revealed that *Kcnn*4^−/−^ mice had enlarged salivary glands with increased collagen deposition (fibrosis) but no major disorganization on H&E staining. mRNA‐seq identified 473 differentially expressed genes (DEGs), with significant enrichment of T‐ and B‐cell‐related pathways. Immunofluorescence showed markedly increased infiltration of CD4^+^, CD8^+^ T cells and CD19^+^ B cells into the salivary glands of knockout mice, yet local IgG levels were significantly decreased, which was confirmed by western blotting. Olink proteomic analysis of serum from *Kcnn*4^−/−^ mice revealed that among 43 targeted inflammatory proteins, 16 were significantly changed; 15 of these (including Il‐6, Tnf, Cxcl1) were decreased, while only Csf1 was increased, indicating a broad systemic anti‐inflammatory shift. Collectively, these findings demonstrate that Kcnn4 deficiency induces salivary gland fibrosis, promotes T/B cell infiltration, impairs local antibody production, and suppresses systemic inflammatory mediators, uncovering an unexpected role of Kcnn4 in integrating tissue remodeling and immune function in the salivary gland.

## Graphical abstract

Kcnn4 deficiency leads to a marked enlargement of salivary glands with increased collagen deposition. Increased infiltration of CD4^+^, CD8^+^ T cells and CD19^+^ B cells into the salivary glands of *Kcnn4−/−* mice. Proteomic profiling of serum inflammatory proteins demonstrates that Kcnn4 ablation broadly reduces the levels of pro‐inflammatory proteins.

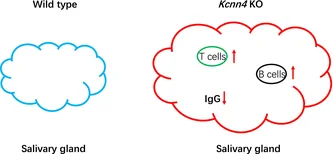

## 1 Introduction

The salivary gland is essential for maintaining oral homeostasis, and its dysfunction—most notably in Sjögren's syndrome or following radiotherapy—leads to hyposalivation, fibrosis, and chronic inflammation (Qi et al. 2024). The underlying molecular mechanisms that govern the balance between immune activation and tissue integrity in salivary glands remain incompletely understood (Porcheri and Mitsiadis 2019; Mahapatra 2025).

Kcnn4 (also known as KCa3.1) encodes a calcium‐activated potassium channel that is widely expressed in both epithelial and immune cells (Fanger et al. 1999; Ghanshani et al. 2000; Srivastava et al. 2005; Wulff et al. 2001). It plays a key role in calcium signaling, cell volume regulation, proliferation, and migration (Ishii et al. 1997; Rapetti‐Mauss et al. 2015; Srivastava et al. 2008). In immune cells, Kcnn4 has been implicated in T‐cell activation (Chiang et al. 2017; Di, Srivastava, Zhdanova, Ding, et al. 2010; Jusztus et al. 2025; Zophel et al. 2023), B‐cell differentiation (Caillot et al. 2018; Zhang et al. 2016), macrophage function regulation (Jiang et al. 2019), and cytokine production (Zhang et al. 2016; Zhao et al. 2025). However, its physiological and pathophysiological functions in the salivary gland have not been systematically explored.

In this study, we generated a constitutive *Kcnn4* knockout mouse model to investigate the impact of Kcnn4 loss on salivary gland homeostasis. We show that Kcnn4 deficiency leads to a marked enlargement of salivary glands with increased collagen deposition (fibrosis), although the overall histoarchitecture remains largely preserved. Transcriptomic analysis reveals that Kcnn4 deletion alters a limited set of genes but significantly enriches pathways related to T and B cell functions. Consistent with this, we observed increased infiltration of CD4^+^, CD8^+^ T cells and CD19^+^ B cells into the salivary glands of knockout mice. Surprisingly, the augmented B‐cell infiltration is accompanied by decreased local IgG levels, suggesting a functional impairment of B cells. Furthermore, proteomic profiling of serum inflammatory proteins demonstrates that Kcnn4 ablation broadly reduces the levels of pro‐inflammatory proteins, indicating a global anti‐inflammatory shift. Collectively, our results reveal an unexpected role of Kcnn4 in suppressing salivary gland fibrosis and modulating immune cell infiltration, and they highlight a previously unrecognized dissociation between B‐cell homing and antibody production upon Kcnn4 loss.

## 2 Methods

### 2.1 Mice Breeding

Heterozygous *Kcnn4* knock out mice (C57BL/6J background) were brought from Cyagen Biosciences company (Suzhou, China). Mice were maintained under standard conditions at Henan Zhongmo Biotechnology Research Institute. All experimental procedures were reviewed and approved by the Animal Ethics Committee of Henan Zhongmo Biotechnology Research Institute (ethics approval no. ZM‐202603014).

### 2.2 H&E Staining and Masson Staining

When mice were 8 weeks old, they were humanely sacrificed and the excised salivary glands fixed with formaldehyde and embedded in paraffin sections. 5 μm paraffin sections of mice salivary glands were stained with H&E staining kit (Beyotime, C0105S) and Masson staining kit (Solarbio, G1346). H&E staining and Masson staining were conducted in strict accordance with the manufacturer's standardized protocol.

### 2.3 Transcriptome Sequencing

Mice salivary glands were collected and isolated total RNA, then constructed library for transcriptome sequencing; this part work was done by General Biol (Anhui) Co. Ltd. The detail method was described in our previous paper (Li et al. 2023; Wang et al. 2026). Differential regulated genes were assigned by applying thresholds of log2FC ≥ 1.0 and log2FC ≤ −1.0 with *p* < 0.05 and presented as a Volcano plot.

### 2.4 Immunofluorescence Staining of Paraffin‐Embedded Tissue Sections

Paraffin‐embedded tissue sections (5 μm thick) were deparaffinized in xylene twice for 10 min each, then rehydrated through a graded ethanol series (100%, 95%, 80%, 70%; 5 min each) and finally rinsed in distilled water. Antigen retrieval was performed by immersing the sections in citrate buffer and heating in a pressure cooker or microwave for 10–15 min. The sections were then allowed to cool slowly to room temperature. After washing three times with phosphate‐buffered saline (PBS, 5 min each), sections were blocked with 5% normal donkey or goat serum in PBS containing 0.3% Triton X‐100 for 60 min at room temperature to reduce non‐specific binding. Sections were incubated with the primary antibody (diluted in blocking buffer) overnight at 4°C in a humidified chamber. After washing three times with PBS (10 min each), sections were incubated with fluorophore‐conjugated secondary antibody for 1 h at room temperature in the dark. Sections were washed three times with PBS (5 min each), then incubated with DAPI (1 μg/mL) for 5 min at room temperature for nuclear staining. Finally, sections were mounted with anti‐fade mounting medium and coverslipped. CD4 (Proteintech, 67786‐1‐Ig), CD8 (Proteintech, 66868‐1‐Ig), CD19 (Proteintech, 27949‐1‐AP).

### 2.5 Western Blotting

Mice salivary gland proteins were extracted by RIPA buffer containing protease inhibitors with tissue grinder (Servicebio, SWE‐3D, China). The detailed western blotting experimental procedures were described in our previous papers (Li et al. 2023; Wang et al. 2024). Primary antibodies information: Kcnn4 (Proteintech, 23271‐1‐AP), Gapdh (Proteintech, 10494‐1‐AP). IgG was detected by anti‐mouse IgG HRP without primary antibody.

### 2.6 Blood Collection, Serum Processing and Olink Proteomics Analysis

Blood samples were obtained by orbital sinus bleeding (5 mice per group). After clotting at room temperature for 30 min, the samples were centrifuged (3000 rpm for 10 min) to isolate serum. The serum samples were then submitted to Shanghai Zhongkepurui (Shanghai, China) for Olink proteomics analysis.

### 2.7 Statistical Analysis

Statistical analyses were performed with GraphPad Prismversion 7.0. All results are presented as mean ± SD of three independent experiments. The unpaired Student's *t*‐test was used for two group comparisons. *p* < 0.05 was considered as statistically significant.

## 3 Results

### 3.1 Knock Out *Kcnn4* Mice Exhibit Volume Increase and Fibrosis of Salivary Glands

We got homozygote *Kcnn4* knock out mice (*Kcnn4* ^*−/−*^), which constitutively deleted exon 4 of *Kcnn4* (Figure 1A), we found *Kcnn4* ^*−/−*^ mice look normal. Knock out *Kcnn4* didn't affect size, body weight (Figure 1B), or fertility of female or male mice, so we dissected *Kcnn4* ^*−/−*^ mice to observe organs, which were relatively normal, except the salivary glands. Salivary glands of *Kcnn4* ^*−/−*^ mice exhibited larger than wild type mice (Figure 1C). H&E staining images of salivary glands sections showed it was not a significant histological difference between *Kcnn4* ^*−/−*^ mice and wild type mice (Figure 1D). While Masson staining of salivary glands sections showed elevated collagen deposition in *Kcnn4* ^*−/−*^ mice than wild type mice (Figure 1E,F).

**FIGURE 1:**
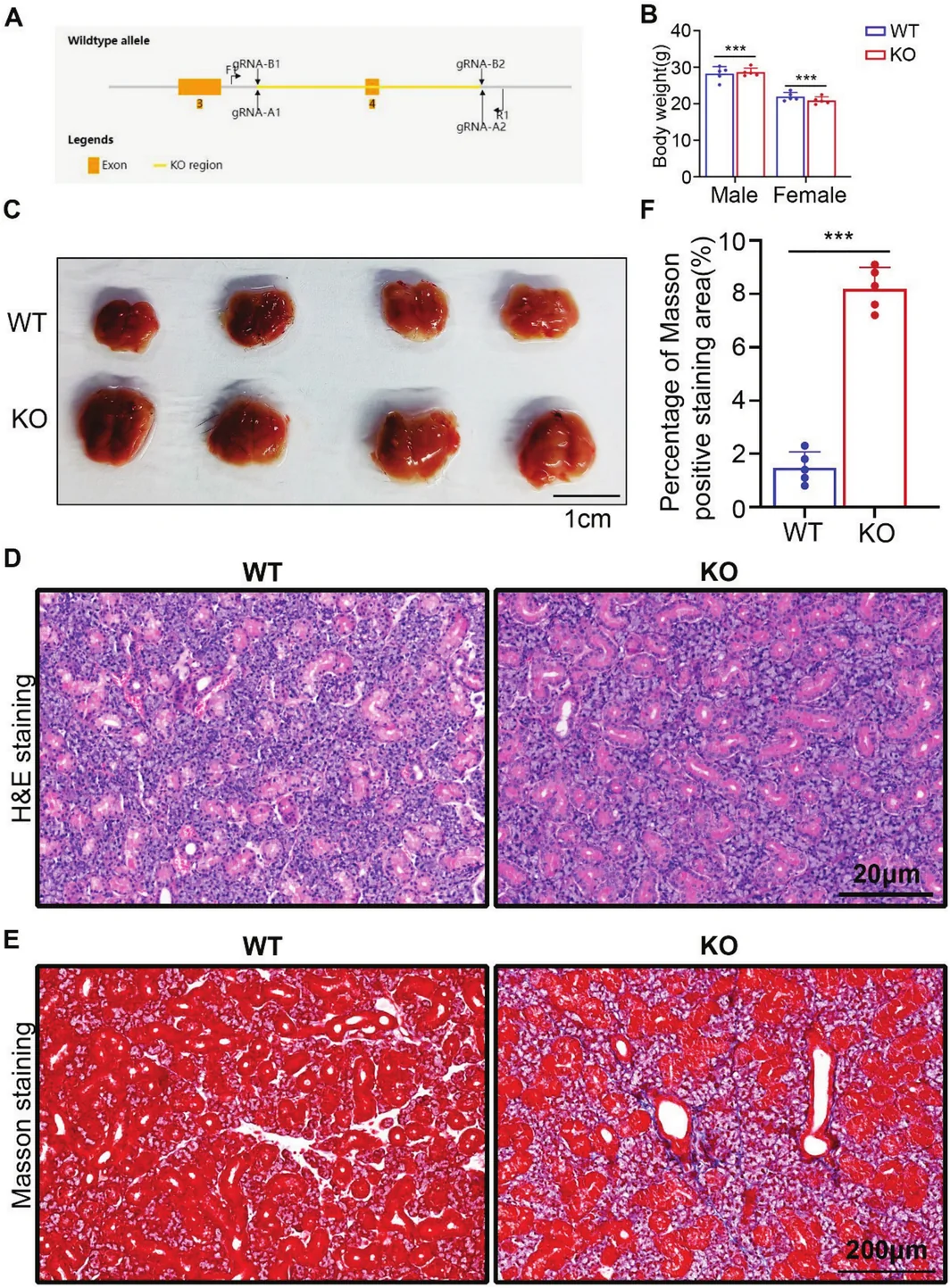
Knock out *Kcnn4* mice exhibit volume increase and fibrosis of salivary glands. (A) Schematic of the strategy for generating *Kcnn4* knockout mice. (B) Body weight of 3 months old *Kcnn4* KO and WT mice. (C) Volumes of salivary glands were increased in *Kcnn4* knockout mice group. (D) No significant histological difference between wild type and *Kcnn4* knockout mice group. (E, F) Representative images of Masson staining using wild type and *Kcnn4* knockout mice salivary glands sections (E) Quantification of Masson staining positive area between two groups, *N* = 5 mice/group (F). Data were presented as mean ± SD, unpaired Student's *t*‐test. ***p < 0.001.

### 3.2 Knock Out *Kcnn4* Slightly Changes Transcriptomics of Salivary Glands

To elucidate the mechanism underlying the observed changes in salivary glands in *Kcnn4* ^*−/−*^ mice, we conduct mRNA‐seq using total RNA extracted from salivary glands of wild type and *Kcnn4* ^*−/−*^ mice (4 mice per group). Differential regulated genes were assigned by applying thresholds of log2FC ≥ 1.0 and log2FC ≤ −1.0 with *p* < 0.05 and presented as a Volcano plot. Volcano map of transcriptome sequencing showed 473 differentially expressed genes after knockout *Kcnn4*, including 308 up‐regulated genes and 165 down‐regulated genes (Figure 2A). Heat map of DEGs also showed (Figure 2B). Gene Ontology and pathways analysis with DEGs indicated many pathways associated with T cells and B cells functions were enriched (Figure 2C,D).

**FIGURE 2:**
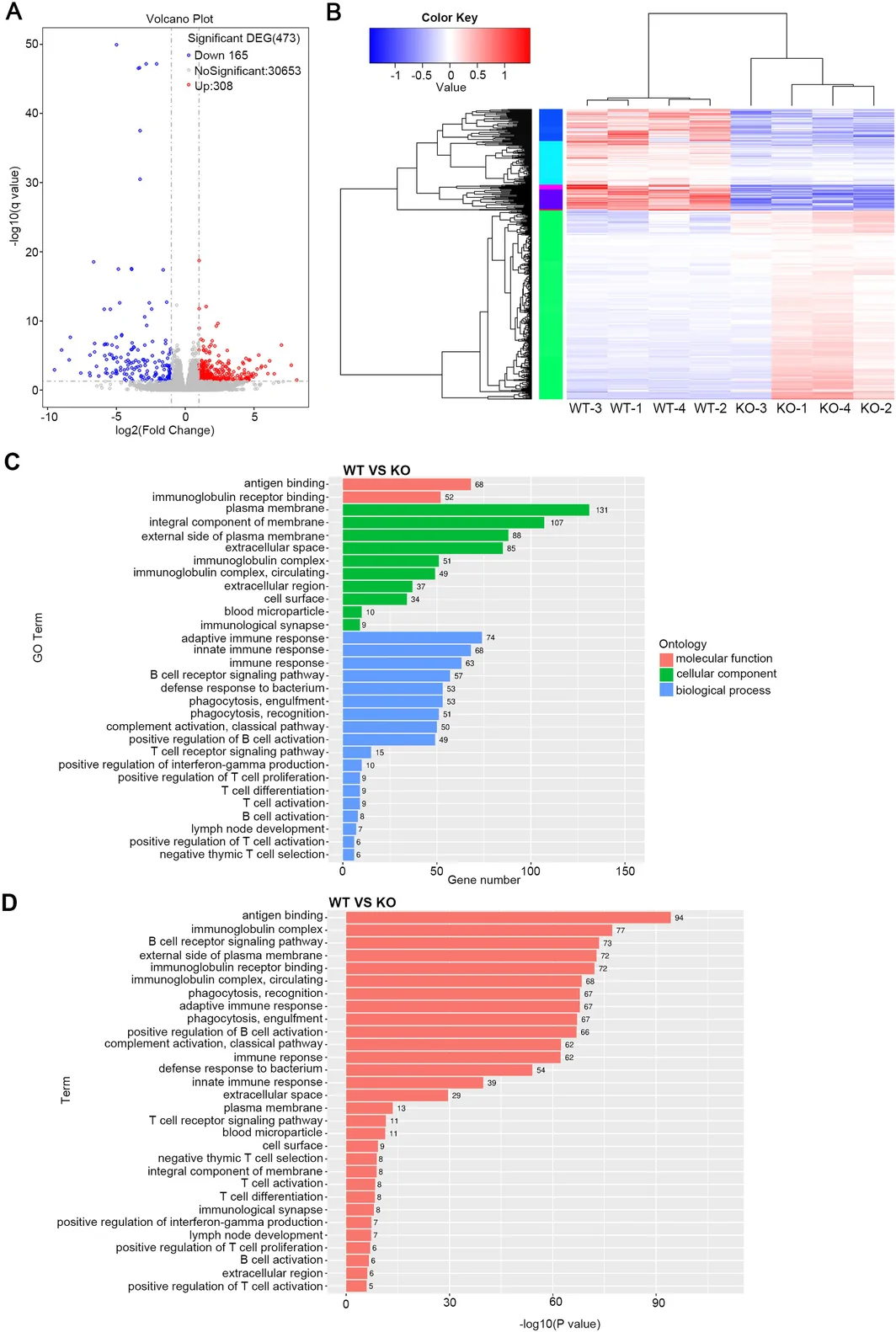
Knock out *Kcnn4* slightly changes transcriptomics of salivary glands. (A) Volcano dot map of mRNA‐seq of salivary glands from 4 wild type male mice and 4 *Kcnn4* knockout mice. (B) Heat map of differentially expressed genes. (C) Gene Ontology analysis of differentially expressed genes. (D) Pathways enriched with differentially expressed genes.

### 3.3 Knock Out *Kcnn4* Promotes T Cells and B Cells Infiltration in Salivary Glands

As many T cells and B cells functions pathways were enriched in mRNA‐seq assay, we detected T cells and B cells infiltration in salivary glands. CD4 and CD8 were used as T cells markers; immunofluorescence staining images and quantification data of positive cells percentage in salivary glands sections showed CD4 and CD8 positive cells were significantly increased in *Kcnn4* knock out mice compared with wild type mice (Figure 3). CD19 was used as B cells marker; immunofluorescence staining images and quantification data of positive cells percentage in salivary glands sections showed CD19 positive cells were significantly increased in *Kcnn4* knock out mice (Figure 4A,C). We also detected IgG levels in salivary glands; immunofluorescence staining images and quantification data of IgG immunofluorescence intensities showed IgG levels were significantly decreased in *Kcnn4* knock out mice (Figure 4B,D).

**FIGURE 3:**
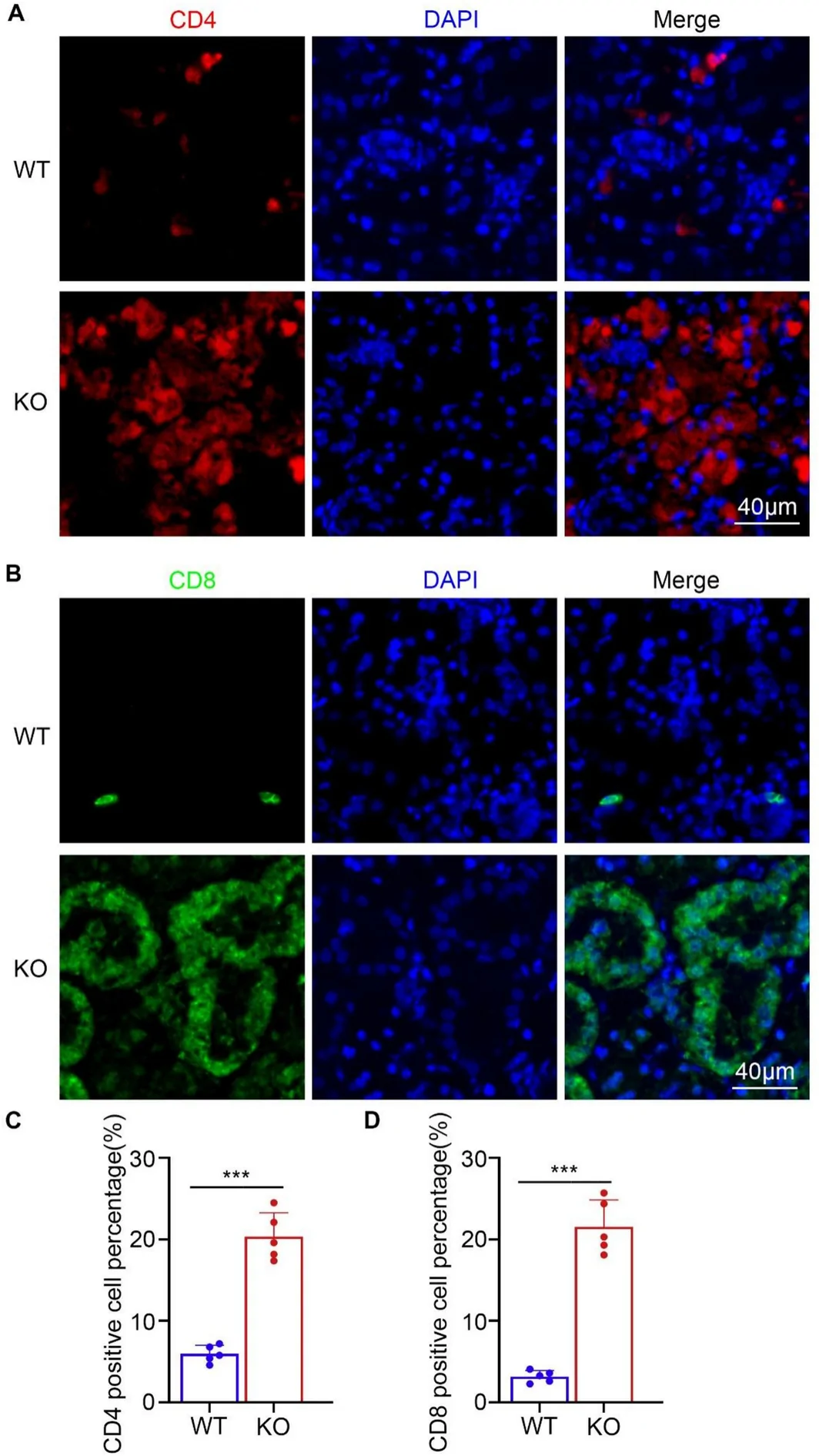
Knock out *Kcnn4* promotes T cells infiltration in salivary glands. (A, B) Immunofluorescence staining of T cell marker CD4 (A) and CD8 (B) using wild type and *Kcnn4* knockout mice salivary glands section. (C, D) Quantification of CD4 (C) and CD8 (D) staining positive cells percentage in wild type and *Kcnn4* knockout mice salivary glands sections. Cell nucleus was stained with DAPI (blue). *N* = 5 mice/group. Data were presented as mean ± SD, unpaired student's t test. ***p < 0.001.

**FIGURE 4:**
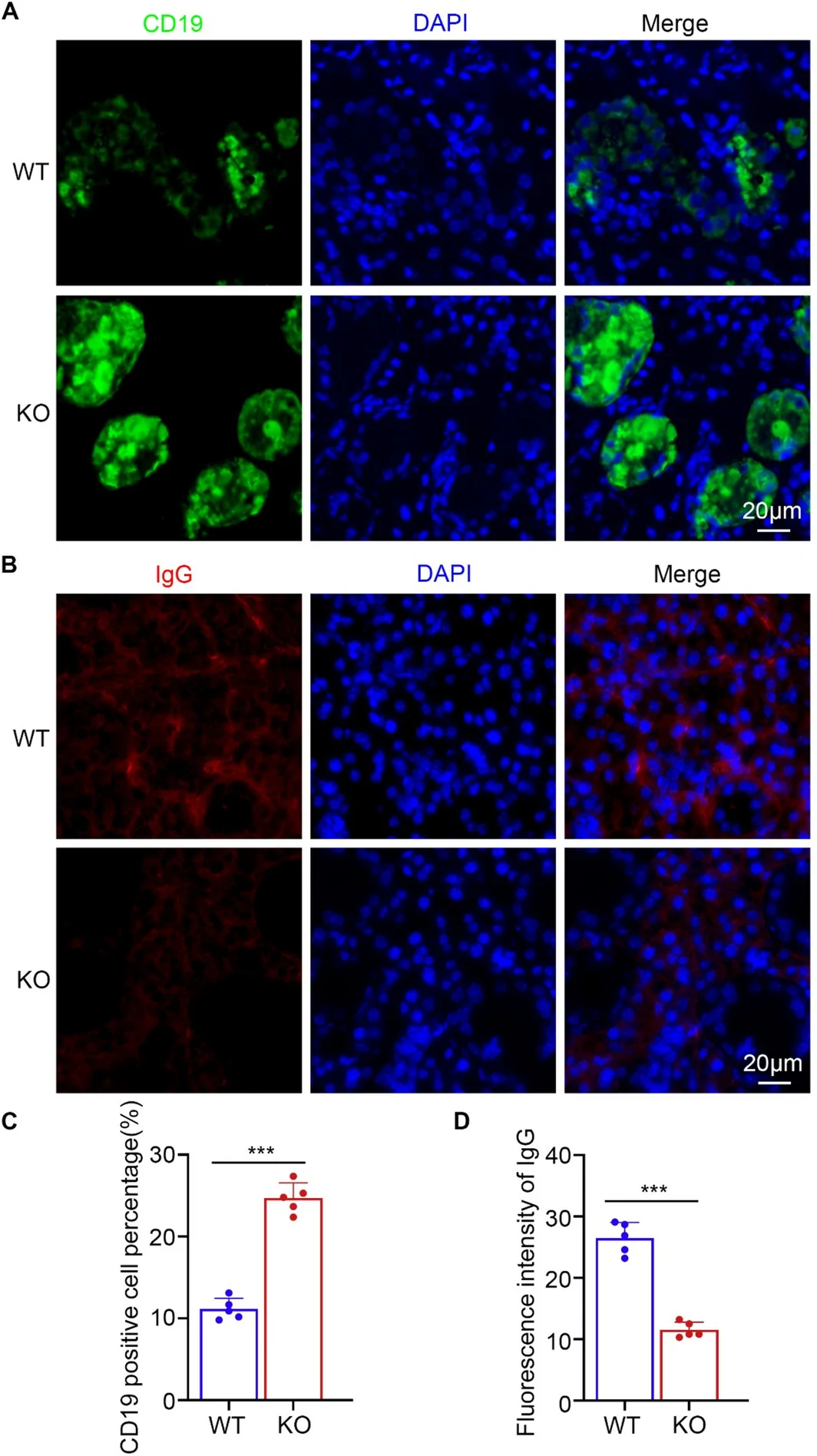
Knock out *Kcnn4* promotes B cells infiltration in salivary glands. (A, B) Immunofluorescence staining of B cell marker CD19 (A) and IgG (B) using wild type and *Kcnn4* knockout mice salivary glands section. (C, D) Quantification of CD19 (C) staining positive cells percentage and IgG intensities (D) in wild type and *Kcnn4* knockout mice salivary glands section. Cell nucleus was stained with DAPI (blue). *N* = 5 mice/group. Data were presented as mean ± SD, unpaired Student's *t* test. ***p < 0.001.

We also extracted proteins from salivary glands of wild type and *Kcnn4* ^*−/−*^ mice (5 mice per group), and conducted western blotting assay; Kcnn4 protein was totally disappeared in KO group (Figure 5A,B). IgG protein levels significantly decreased in KO group (Figure 5C,D), which is consistent with immunofluorescence staining results (Figure 4B,D). More B cells infiltration with decreased IgG levels indicates dysfunction of B cells in salivary glands of *Kcnn4* knock out mice.

**FIGURE 5:**
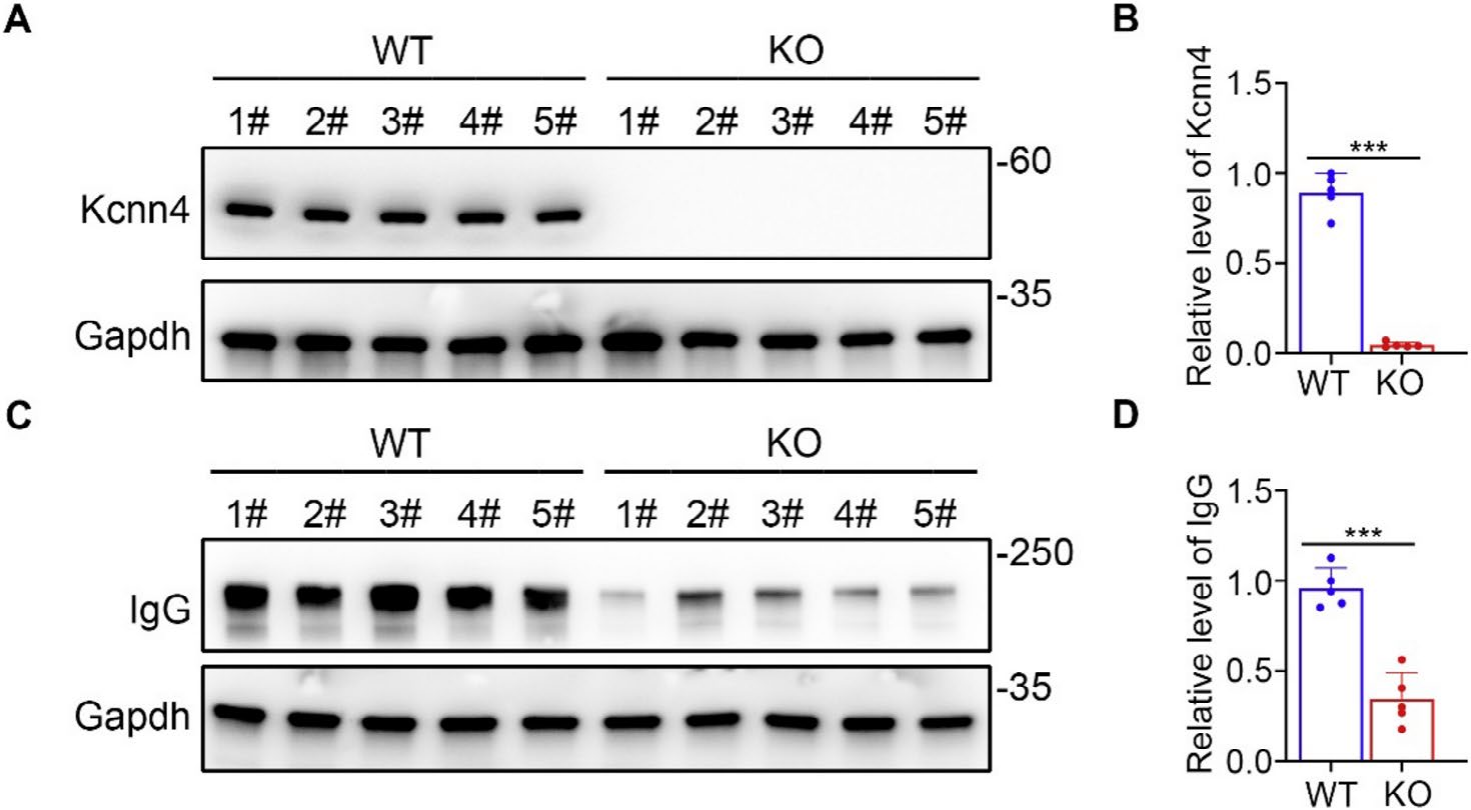
IgG levels were decreased in salivary glands of *Kcnn4* knockout mice. (A, C) Kcnn4 (A) and IgG (C) levels in mice salivary glands were detected by western blotting, Gapdh served as loading control. (B, D) Quantification of Kcnn4 (B) and IgG (D) levels in wild type and *Kcnn4* knock out mice salivary glands. *N* = 5 mice/group. Data were presented as mean ± SD, unpaired student's t test. ***p < 0.001.

### 3.4 Knock Out *Kcnn4* Decreases Inflammatory‐Related Proteins Levels in Mice Serum

To investigate the global inflammatory reaction after knock out *Kcnn4* in mice, we employed Olink proteomics technology to detect 43 targeted inflammatory‐related proteins in mice serum. Principal component analysis map of inflammatory‐related protein levels in serum of two groups of mice (5 mice per group) showed knock out *Kcnn4* changed inflammatory‐related protein levels in mice serum (Figure 6A). Among the targeted 43 proteins in the Olink panel, 16 protein levels were significantly changed, 25 protein levels were not significantly changed, and another 2 proteins were not detected (Figure 6B). Heat map of all 41 inflammatory‐related protein levels that were detected in serum of two groups of mice was shown (Figure 6C). Heat map of 16 significantly changed inflammatory‐related proteins also showed, just Csf1 levels were significantly increased in serum of KO group (Figure 7P), other 15 proteins were significantly decreased in serum of KO group (Figures 6D and 7A–O). These Olink proteomics data indicated that knock out *Kcnn4* inhibited inflammatory reaction in mice.

**FIGURE 6:**
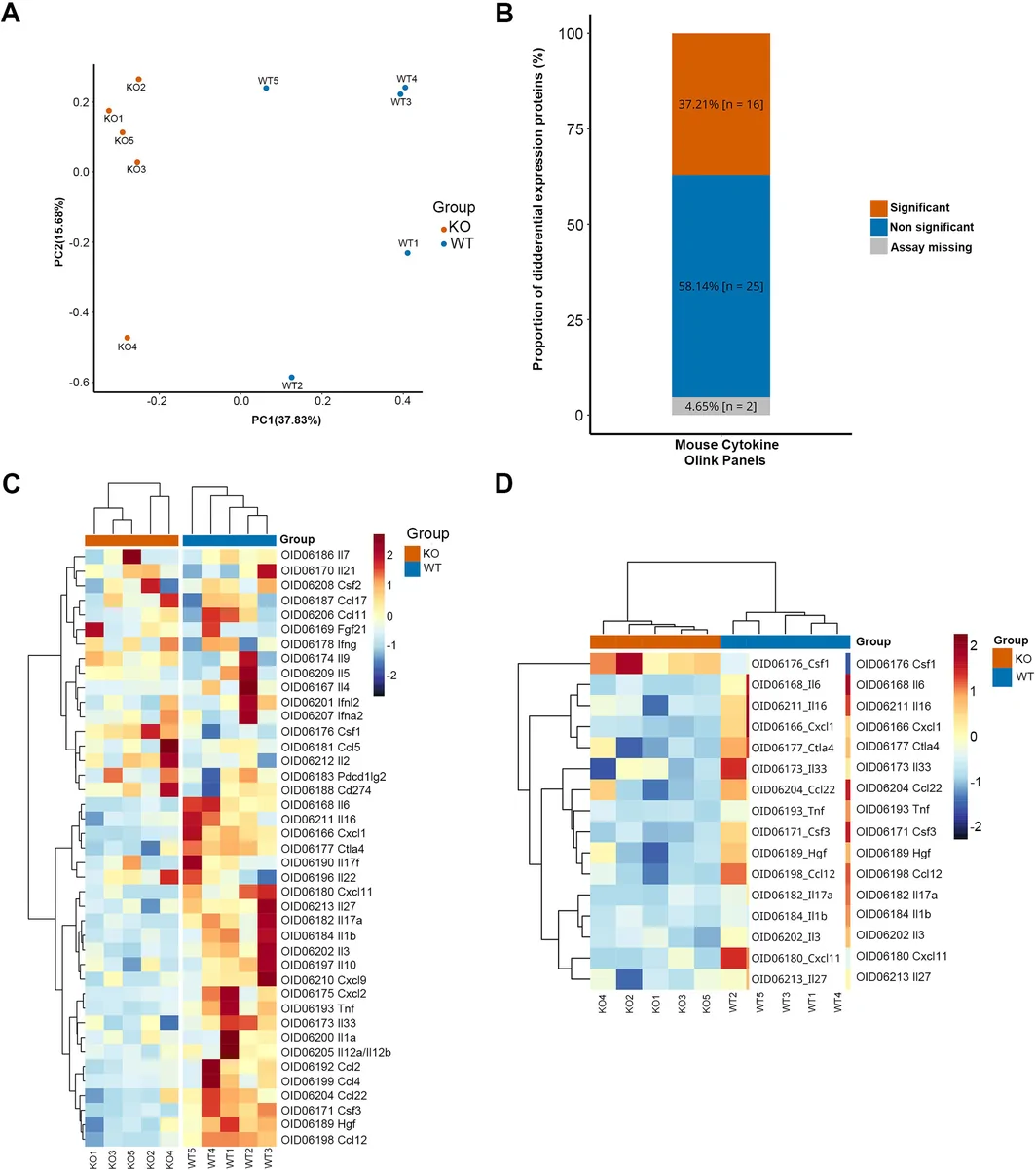
43 inflammatory‐related proteins in *Kcnn4* knockout mice serum were detected by Olink proteomics technology. (A) Principal component analysis map of inflammatory‐related protein levels in serum of wild type and Kcnn4 knockout mice. (B) Proportion of differential expression proteins between two groups. (C) Heat map of all 43 inflammatory‐related proteins in two group mice serums. (D) Heat map of 16 differently expressed inflammatory‐related proteins in two group mice serums. *N* = 5 mice/group. Unpaired Student's *t* test. *p* < 0.05 means significant difference.

**FIGURE 7:**
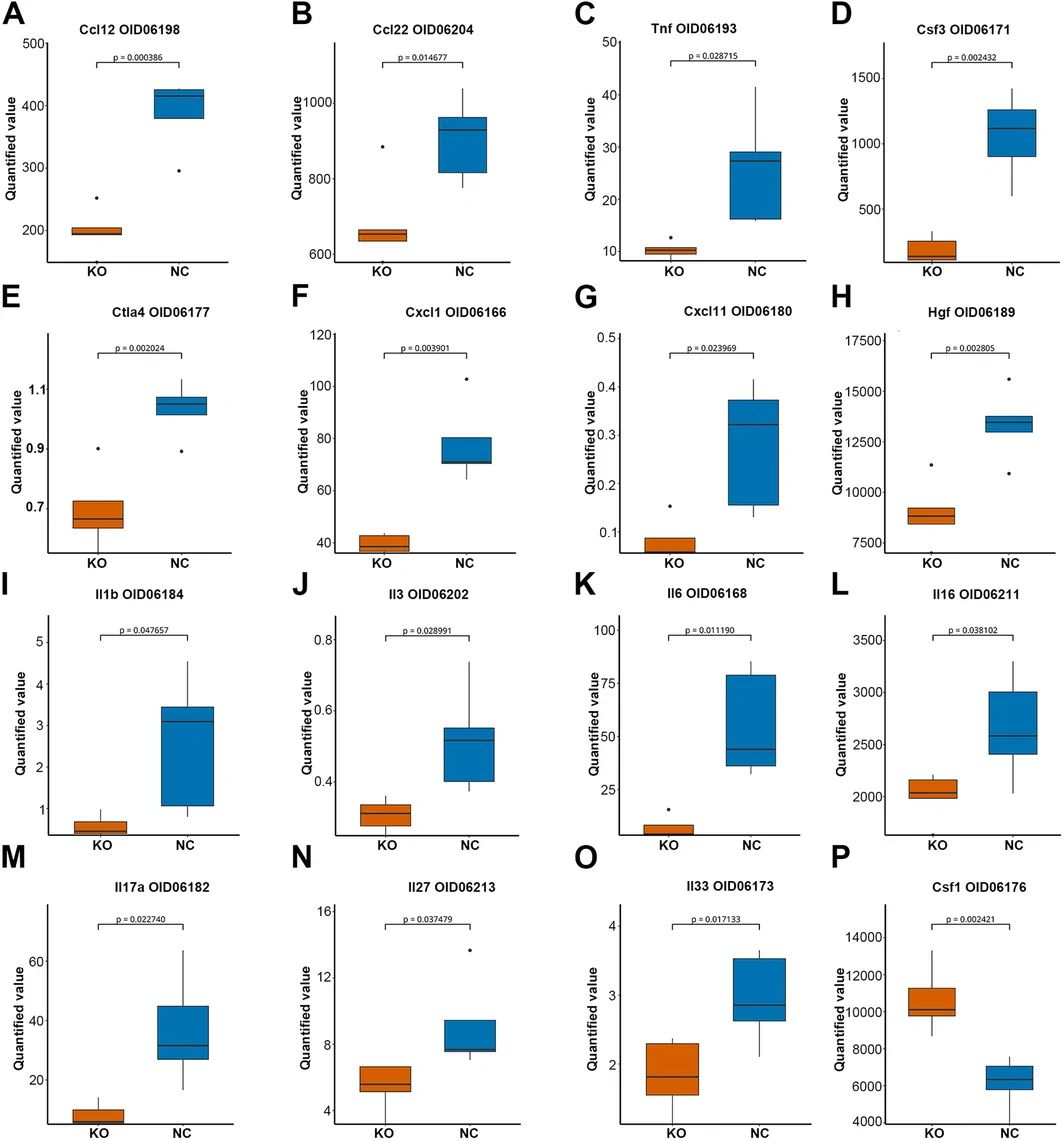
16 inflammatory‐related proteins differentially expressed in *Kcnn4* knockout mice serum. (A–O) 15 inflammatory‐related proteins were downregulated in Kcnn4 knockout mice serum, including Ccl12 (A); Ccl22 (B); Tnf (C); Csf3 (D); Ctla4 (E); Cxcl1 (F); Cxcl11 (G); Hgf (H); Il1b (I); Il3 (J); Il6 (K); Il16 (L); Il17a (M); Il27 (N); Il33 (O). (P) Csf1 was upregulated in Kcnn4 knockout mice serum.

## 4 Discussion

In this study, we demonstrate that constitutive knockout of Kcnn4 in mice results in a unique salivary gland phenotype characterized by organ enlargement, mild fibrosis, increased infiltration of T and B lymphocytes, yet a paradoxical decrease in local IgG levels. Moreover, systemic inflammatory protein profiling reveals a widespread reduction of pro‐inflammatory proteins. Collectively, our data suggest that Kcnn4 plays a complex, context‐dependent role in salivary gland immunity and tissue remodeling.

Although H&E staining did not reveal overt architectural disruption, Masson staining showed significantly elevated collagen deposition in Kcnn4^−/−^ salivary glands. This finding indicates that Kcnn4 may normally suppress fibrotic processes. Kcnn4 has been implicated in fibroblast proliferation and myofibroblast differentiation in other tissues (Mann et al. 2019; Yao et al. 2025), and its loss might therefore promote extracellular matrix accumulation. However, the absence of major structural changes suggests that the fibrotic response is mild or compensated in this model.

One of the most striking observations is the simultaneous increase in CD4^+^, CD8^+^ T cells and CD19^+^ B cells in knockout salivary glands, accompanied by a sharp reduction in local IgG levels. This dissociation between immune cell homing and effector function is unexpected. Kcnn4 is known to regulate calcium flux and downstream signaling in lymphocytes (Srivastava et al. 2006, 2008). Kcnn4 contributes to activation and proliferation in T cells (Di, Srivastava, Zhdanova, Sun, et al. 2010; Srivastava et al. 2006, 2008); while its role is less characterized in B cells. Our data raise the possibility that Kcnn4 is required for proper B‐cell differentiation into antibody‐secreting plasma cells, even when B‐cell trafficking to the target tissue is enhanced. The reduced IgG levels in both salivary gland homogenates and tissue sections argue against a simple dilution effect, pointing instead to an intrinsic B‐cell defect.

The Olink serum analysis revealed that 15 out of 16 significantly changed inflammatory proteins were decreased, with only Csf1 being elevated. The broad reduction of cytokines such as Il‐6 and Tnf suggests that Kcnn4 ablation suppresses systemic inflammation. This is intriguing because local tissue fibrosis and lymphocyte infiltration might be expected to drive a pro‐inflammatory response. One interpretation is that Kcnn4 in immune cells (or other peripheral tissues) normally supports low‐grade inflammatory tone, and its loss results in an overall “hypo‐inflammatory” state. The increased Csf1 may represent a compensatory attempt to recruit macrophages, but the functional relevance remains to be tested.

Sjögren's syndrome is an autoimmune disorder characterized by lymphocytic infiltration of salivary and lacrimal glands, often leading to hyposalivation and glandular fibrosis (Qi et al. 2024). Interestingly, our Kcnn4 knockout mice exhibit some features reminiscent of Sjögren's (lymphocyte infiltration, fibrosis) but differ in that they have decreased rather than increased IgG and systemic inflammation. This suggests that Kcnn4 loss does not simply recapitulate an autoimmunity model; instead, it may reveal a regulatory node that uncouples infiltration from effector function. Whether Kcnn4 polymorphisms or altered expression contribute to human salivary gland disorders warrants investigation.

Several limitations should be acknowledged. First, salivary gland function was not assessed. We did not measure saliva flow rates, composition (e.g., total protein, amylase, or electrolyte concentrations), or the response to secretagogues. Therefore, whether the observed morphological and immunological changes translate into hypofunction or dysregulation of saliva secretion remains unknown. Second, inflammatory cytokines/chemokines within the salivary gland tissue were not directly measured. Although our serum Olink proteomics provides systemic information, local tissue levels of key inflammatory mediators (e.g., Il‐6, Tnf, Cxcl11) may differ significantly from those in circulation and are critical for understanding the tissue microenvironment. Third, the functional status of infiltrating immune cells in the salivary glands was not analyzed beyond surface marker detection. We identified increased CD4^+^, CD8^+^ T cells and CD19^+^ B cells within the salivary glands by immunofluorescence, but we did not assess their activation state (e.g., T cell exhaustion or anergy, B cell class‐switch recombination, plasma cell differentiation, or cytokine production within the gland). It is therefore unclear whether these infiltrating cells are pro‐inflammatory, regulatory, or functionally impaired. We didn't detect the proportions of circulating immune cells (e.g., CD4+, CD8+, B cells, monocytes) are altered in Kcnn4−/− mice. To address these gaps, it will require additional experiments, such as saliva collection, multiplex cytokine assays on salivary gland lysates, flow cytometry or single‐cell RNA‐seq of isolated glandular immune cells, and functional B‐cell/T‐cell assays.

Additionally, this study uses a constitutive whole‐body knockout, which does not distinguish between cell‐type‐specific effects. Conditional knockout mice (e.g., using *Kcnn4* ^fl/fl^ crossed with epithelial‐ or lymphocyte‐specific Cre lines) are needed to dissect the cellular origin of the phenotype. Finally, the Olink panel focused on 43 predefined inflammatory proteins; unbiased proteomics or cytokine arrays could reveal additional changes.

## 5 Conclusion

In summary, our results identify Kcnn4 as a previously unrecognized regulator of salivary gland fibrosis, lymphocyte infiltration, and antibody production. Loss of Kcnn4 leads to tissue enlargement with mild fibrosis, increased T/B cell homing, local IgG deficiency, and a systemic anti‐inflammatory shift. Future work that directly evaluates salivary gland function, local inflammatory profiles, and immune cell functional status will be essential to fully understand the mechanisms by which Kcnn4 integrates tissue remodeling and immune responses in the salivary gland.

## Author Contributions

**Yuan Zhang:** conceptualization, investigation, methodology, funding acquisition. **Siming Zheng:** investigation, formal analysis. **Degui Jiang:** investigation, visualization. **Cuiyun Cui:** investigation, formal analysis. **Xuan Zhang:** investigation, visualization. **Hanlin Tao:** investigation. **Gongbo Fu:** supervision, writing – original draft. **Linna Cheng:** supervision, writing – original draft. **Shichao Duan:** conceptualization, writing – original draft, investigation, writing – review and editing.

## Funding

This study was supported by Henan Medical Science and Technology Research Project (LHGJ20250094) and Henan Science and Technology Research Project (252102310141).

## Ethics Statement

Mice experiment has been approved by the Animal Ethics Committee of Henan Zhongmo Biotechnology Research Institute (ethics approval no. ZM‐202603014).

## Consent

The authors have nothing to report.

## Conflicts of Interest

The authors declare no conflicts of interest.

## Data Availability

The data that support the findings of this study are available on request from the corresponding author. The data are not publicly available due to privacy or ethical restrictions.
